# Atypical Time to Contact Estimation in Young Adults with Autism Spectrum Disorder

**DOI:** 10.1007/s10803-024-06352-z

**Published:** 2024-04-18

**Authors:** Roberto Vagnetti, Michele Vicovaro, Andrea Spoto, Luca Battaglini, Margherita Attanasio, Marco Valenti, Monica Mazza

**Affiliations:** 1 Department of Biotechnological and Applied Clinical Sciences, University of L’Aquila, L’Aquila, Italy; 2 Department of General Psychology, University of Padua, Padua, Italy; 3 Regional Reference Centre for Autism (Centro di Riferimento Regionale per l’Autismo), Abruzzo Region Local Health Agency 1 (ASL 1), L’Aquila, Italy

**Keywords:** Autism spectrum disorder, Motion extrapolation, Time to contact, Biological motion, Motion perception.

## Abstract

Individuals with Autism Spectrum Disorder (ASD) present atypical sensory processing in the perception of moving stimuli and biological motion. The present study aims to explore the performance of young adults with ASD in a time to contact (TTC) estimation task involving social and non-social stimuli. TTC estimation involves extrapolating the trajectory of a moving target concealed by an occluder, based on the visible portion of its path, to predict the target’s arrival time at a specific position. Sixteen participants with a diagnosis of level-1 ASD (M = 19.2 years, SE = 0.54 years; 3 F, 13 M) and sixteen participants with TD (M = 22.3 years, SE = 0.44 years; 3 F, 13 M) took part in the study and underwent a TTC estimation task. The task presented two object types (a car and a point-light walker), different object speeds, occluder lengths, motion directions and motion congruency. For the car object, a larger overestimation of TTC emerged for ASDs than for TDs, whereas no difference between ASDs and TDs emerged for the point-light walker. ASDs exhibited a larger TTC overestimation for the car object than for the point-light walker, whereas no difference between object types emerged for TDs. Our results indicated an atypical TTC estimation process in young adults with ASD. Given its importance in daily life, future studies should further explore this skill. Significant effects that emerged from the analysis are discussed.

Autism Spectrum Disorder (ASD) is a heterogeneous neurodevelopmental disorder that could affect around the 1% of the population (Valenti et al., 2019). It is characterized by a range of deficits in the domain of social communication and interactions, as well as by repetitive patterns of behavior (American Psychiatric Association, 2013).

Although social deficits appear to represent the core symptomology of ASD, there is now a growing body of evidence showing that unusual sensory processing represents a possible cause of many of the signs and behavioral symptoms of ASD (Simmons et al., 2009). Atypical sensory processing can be detected as early as 6 months of age in children who later receive an ASD diagnosis (Baranek et al., 2013), and could predict the core symptoms associated with the disorder (Boyd et al., 2010; Turner-Brown et al., 2013). ASDs might exhibit a specific sensory processing style, with a bias in processing local details of a stimulus rather than processing a global pattern (Lebreton et al., 2021), which might be associated with the talents they may display (Baron-Cohen et al., 2009). This could represent a situational advantage in visual search paradigms (Kéïta et al., 2010), as some evidence indicates that ASDs may perform better than typical development (TD) controls in these tasks (O’Riordan et al., 2001; Plaisted et al., 1998). Magnetoencephalography recordings have indicated that, with respect to TDs, ASDs might have better access to early visual brain processes (Falter et al., 2011) and might exhibit greater temporal resolution in the visual domain (Falter et al., 2012). Better temporal visual acuity may emerge as an adaptive consequence to impairments in other types of temporal processing (Allman, 2015). However, processing of visual stimuli is closely related to the required attentional focus, and ASDs show a slowdown in attention orientation when faced with a broad attentional focus (Ronconi et al., 2018).

Many perceptual and attentional difficulties related to the disorder are also reported. Literature indicated that ASDs could exhibit impairments regarding spatial attention (Keehn et al., 2013), which is present even early in life (Sacrey et al., 2014). They could present an impaired perception of moving stimuli (Dakin & Frith, 2005), an altered low-level processing of stimulus features such as contrast, orientation, and spatial frequency (Bertone et al., 2005), and impairments related to the processing of global motion (Vandenbroucke et al., 2008). Moreover, they could present difficulties in combining different types of perceptive information (Feldman et al., 2018), an aspect related to their atypical temporal synchrony (Murat Baldwin et al., 2021; Ronconi et al., 2022; Stevenson et al., 2014). In a study by Whyatt and Craig (2013), children with ASD were required to catch a ball going down a ramp, and results showed that ASDs had lower performance than control groups, indicating reduced spatiotemporal control.

## Biological Motion Perception in ASD

Literature has extensively focused on the perception of biological motion (BM) in ASD, which is a prerequisite for the ability to make inferences about the actions of other humans or animals from the way they move (Troje, 2008).

One stimulus widely used to study human BM is the point-light walker (PLW), which consists of moving bright dots representing a walking person in a degraded form (Inuggi et al., 2018). With respect to TDs, ASDs generally present lower performances in the perception and in the interpretation of BM, and difficulties in extracting information from BM have negative effects on social functioning (Pavlova, 2012). ASDs also exhibit impairments in the processing of social information gained from BM (Simmons et al., 2009). Understanding bodily signals represents an important element within social cognition (Pavlova, 2012) as it allows one to extract others’ intentions and to predict their behavior (Blakemore & Decety, 2001). It is known that ASDs present impairments in social cognition (Baron-Cohen et al., 1985; Happè et al., 2017; Pino et al., 2020) and exhibit an altered communication between its domains (Vagnetti et al., 2020). Difficulties experienced by ASDs tend to increase when BM is used for other purposes, such as inferring intentionality or emotions, while difficulties tend to decrease with the increase of age (Federici et al., 2020; Todorova et al., 2019). Accordingly, one study indicated that adults with ASD could achieve performances comparable to those of a control group when they were requested to process human movements, however they utilized a different brain network (McKay et al., 2012). In other words, ASDs can achieve levels of performance comparable to those achieved by neurotypical controls on tasks related to biological motion, though the underlying neural process might be different (Freitag et al., 2008). In their study, Karaminis and collaborators (2020) combined a speed discrimination task with BM, with the aim to compare TD’s and ASDs’ adaptation to visual speed, and found similar adaptation effects in both groups (but see van Boxtel et al., 2016).

## Time to Contact Estimation

The present study explores a specific perceptual-cognitive ability, that is the estimation of the time it takes for a moving object to reach a stationary target after it becomes hidden from view. Given that many objects in our visual field can be partially or completely obscured by obstacles, the ability to estimate the position of concealed moving objects can be crucial to avoid collisions with them, such as anticipating when a car will reappear from behind a wall. This topic has a long history; for example, in the 1950s, researchers focused on studying how factors like speed, acceleration, and distance impacted participants’ abilities in tasks that required them to maintain a representation of the motion behavior of occluded objects (Gottsdanker, 1956; Slater-Hammel, 1955; Wiener, 1962). In the era of the cold war, these researches were relevant in understanding how aircraft gunners could improve their tracking of enemies behind clouds (Gottsdanker, 1952).

State-of-the-art experiments typically involve a task known as *prediction motion*. In this task, participants are presented with a moving object that, at some point in its trajectory, passes behind a visible or invisible occluder. The participants’ objective is to press a button when the occluded object would reach a target position (Makin, 2018; Rosenbaum, 1975). Some authors have referred to the perceptual-cognitive operation behind this task as *motion extrapolation* (Battaglini & Ghiani, 2021; Yakimoff et al., 1993) or simply *extrapolation* (Jagacinski et al., 1983). Drawing an analogy from mathematics, where extrapolation means estimating values beyond the known dataset, this term describes the observer’s task of estimating the position of a moving object beyond the available sensory data. However, instead of using terms like motion extrapolation or prediction motion, which may reflect theoretical assumptions, we employ the more specific and concrete term like time to contact (TTC) estimation.

In a TTC estimation task, the difference between the real and the estimated arrival time (or the opposite) is the timing (or constant) error (Sokolov & Pavlova, 2003). Several strategies have been proposed by which individuals would estimate TTC (for a recent review see Battaglini & Ghiani, 2021). For instance, as suggested by Makin (2017), participants may rely on a mental simulation of the object’s motion, updated through a control mechanism, which can be common for different dimensions (i.e., the common rate control hypothesis), or separate for each dimension (i.e., the separate rate control hypothesis). Another proposed strategy is called the clocking strategy, by which individuals would estimate the time needed to the object to reach a target position before the occlusion, and then they would count down to provide the correct answer (DeLucia & Liddell, 1998; Tresilian, 1995). Several factors can influence performance in TTC tasks, such as physical features of the target, textures utilized, presence of distractors, and the attention deployed to the task (Battaglini & Ghiani, 2021). TTC tasks can provide insights into how we process our environment and into our representations of the physical behavior of objects (Battaglini & Mioni, 2019; Makin et al., 2009).

Neuroimaging evidence has evaluated the cortical areas involved during tasks that require the updating of the spatial position of occluded moving targets. Many areas are active during occlusion, including the cerebellum, areas related to the visual field, basal ganglia, and premotor cortex (Lencer et al., 2004). Jiang and collaborators (2008) asked participants in their study to track a moving stimulus during occlusion and indicated activity in the right visual cortex. O’Reilly et al. (2008) suggested that the cerebellum could be involved in the timing of events when a model of change over time is required, and then the temporal prediction would be used to set other cortical areas involved in spatial prediction. Besides the cerebellum, other regions are probably involved in estimation of the occluded position of hidden moving targets, and basal ganglia and dorsal striatum seem to play a crucial role (Coull et al., 2011). Notably, ASDs present alterations in the cerebellum and basal ganglia (Becker & Stoodley, 2013; Subramanian et al., 2017).

To our knowledge, TTC estimation has never been explored in the context of autism to date. This ability can have importance in daily life as we often deal with moving objects. It can be especially important for road safety, considering that the motion of cars and pedestrians can be frequently occluded by various obstacles. Driving requires an accurate motion perception, which plays a fundamental role in tasks such as detecting pedestrian incursions (Straughn et al., 2009) or controlling others’ speed while entering a curve (Wilkie & Wann, 2003). Although driving involves many high-level cognitive skills, accurate motion perception and extrapolation are fundamental for crash risk reduction (DeLucia et al., 2003). Being able to drive represents improvements in individual’s independence and self-esteem, enhancing employment, vocational, and social opportunities (Ekelman et al., 2009). However, ASDs can face many difficulties in acquiring a driver’s license, and only 24% of adults with ASD can obtain it, compared to the 75% of the general population (Feeley, 2010; Vindin et al., 2021). In the present study, we aim to compare the performance of a sample of TD individuals and a sample of ASD individuals in a TTC estimation task.

## Outline of the Present Study

A group of young adults diagnosed with ASD and a control group of TD were subjected to a TTC estimation task characterized by two types of objects, one representing a point-light walker (PLW), and the other representing a physical inanimate object (i.e., a car). On each experimental trial, the participant was presented with one of the two objects (i.e., PLW or car) moving horizontally on a computer screen at a constant speed. At some point of its trajectory, the stimulus disappeared behind an invisible occluder. The participant’s task was to press a key when the stimulus reached a target position represented by a visible vertical bar. The timing error (TE) was measured, defined as the difference between the estimated and the real physical TTC. A positive TE indicates an overestimation of TTC.

We tested the following two hypotheses: (1) Based on evidence that suggests that ASD individuals face a variety of impairments in motion perception and visual attention compared with TD individuals, ASD individuals might be overall less accurate in TTC estimation with respect to TD individuals. (2) Due to the well-documented impairments of ASD individuals in the processing of biological motion, differences in performance between ASD and TD individuals might be even stronger in the case of the estimation of the TTC of the PLW. ASDs’ specific difficulties with biological motion processing would add to more general deficits in motion perception and visual attention.

For the sake of generality, besides the object type (car or PLW), we manipulated the speed of the object (low or high), the occluder length (short or long) and the congruency of the stimulus orientation with respect to the motion direction (congruent or incongruent). In the congruent trials, the ‘front’ of the stimulus was oriented consistently with the motion direction (i.e., the car appeared to be moving forward and the PLW appeared to be walking forward). By contrast, in the incongruent trials, the stimulus was oriented in the direction opposite to that of motion, therefore the car appeared to be moving in reverse and the PLW moved in the direction opposite to that of walking (i.e., as if walking on a conveyor belt moving backwards). We did not have specific a-priori hypotheses about how object speed, occluder length, and congruency would interact with object type and group. The manipulation of these factors was mainly aimed to provide generalizable results and to decrease the repetitiveness of the stimuli.

## Methods

*Participants*. A total of 16 participants with a diagnosis of level-1 ASD (3 F, 13 M; mean age = 19.2 years, *SE* = 0.54 years; mean RSPM IQ = 106.2, SD = 11.9) and a total of 16 TD participants (3 F, 13 M; mean age = 22.3 years, *SE* = 0.44 years) took part in the experiment on a voluntary basis. ASD participants were recruited from the Reference Regional Centre for Autism (CRRA) in L’Aquila. A diagnosis of level-1 ASD was made by experienced psychiatrists and psychologists following the criteria of the DSM-5 (American Psychiatric Association, 2013) and by the Autism Diagnostic Observation Schedule-2 (ADOS-2; Lord et al., 2012). The ADOS-2 scores of all ASD participants were above the cut-off (mean *Communication and social interaction* = 12.3, SD = 4.1; mean *Stereotyped behaviors and restricted interests* = 0.50; SD = 0.5; mean total score = 12.8, SD = 3.8). For ASD participants, exclusion criteria were: (a) cognitive impairment; (b) the presence of comorbidity; (c) the presence of drug treatment. TD participants were undergraduate students attending psychology courses, recruited from the University of L’Aquila, who were excluded from the study in case of a history of neurological disease, psychiatric disorders, substance disorders, head trauma, or cognitive impairment. Participants’ cognitive skills were assessed through Raven’s Standard Progressive Matrices (RSPM; Raven & Court, 1938), a measure of general intelligence (Raven et al., 2000) that has little dependence on language abilities. The RSPM was used to exclude participants with cognitive impairment who could have difficulties in understanding the proposed task; we excluded participants with a medium-low or lower performance (i.e. < 25th percentile; Raven, 2008) accordingly. The brevity of the RSPM prevented participant fatigue. All the participants were native Italian speakers and gave written informed consent to participate.

*Stimuli and Apparatus*. The participants were seated in a dark room, 57 cm from the display screen. The viewing was binocular. Stimuli were generated with MATLAB and the Psychophysics Toolbox (Brainard, 1997; Pelli, 1997), and were displayed on a notebook HP Pavillon 15, with a 15.6” monitor, and with a refresh rate of 60 Hz. The screen resolution was 1920 × 1080 pixels.

Two object types were used, namely the schematic picture of a white car and a PLW. The car picture was embedded in an invisible rectangle of 4.5 × 1.6 deg (length of the car picture: 4.5 deg). The PLW was built by adapting the code used in Mather et al. (2016), which in turn was based on the algorithm described in Cutting (1978). The shifting pattern of dots generated by a step cycle of a walking figure was sampled to create forty static views. The static views were presented for 33.34 ms each. When this series of static frames was presented in rapid succession, observers reported a compelling impression a walking figure, as expected. Thirteen points were plotted in each frame to define the figure (signal); one for the head, two each (left and right) for the shoulders, elbows, wrists, hips, knees, and ankles. Dot size was 5 pixels (0.08 deg). They simulated the pattern generated by a sideways view of a person walking in horizontal forward motion. The dot displacements contained elliptical and oscillatory components. The walker’s torso was 1.43 deg and the height of the entire figure was 4 deg. The maximum width reached by the walker was 2.15 deg.

**Fig. 1:**
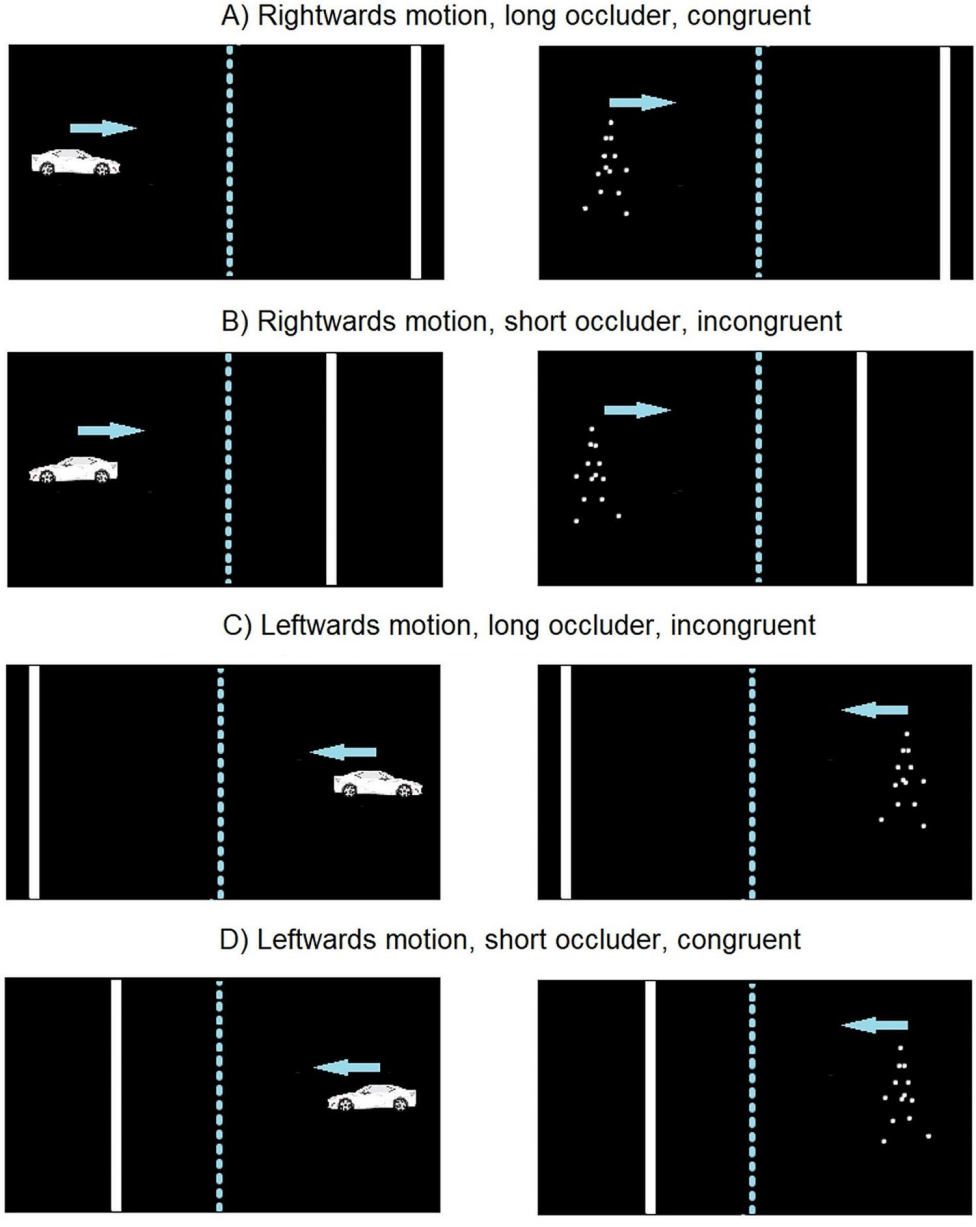
Representation of the stimuli (not drawn to scale). Blue arrows were added in this figure for illustrative purposes, to indicate the motion direction of the target object (car or PLW). The vertical blue dashed lines were also added for illustrative purposes, and indicate the margin of the invisible occluder. The white vertical bars were visible in the original stimuli, and mark the end of the occluder (i.e., the target position)

The car and the PLW were seen in front of a black background. On each trial, the center of the object (i.e., the center of the rectangle embedding the car stimulus and the center of the walker’s torso) appeared 5.35 deg to the left or right from the center of the screen, depending on whether the motion direction was rightwards or leftwards, respectively. The center of the object travelled 7.15 deg before disappearing behind an invisible occluder. On different trials, the car and the PLW could be oriented congruently with the motion direction or incongruently with it (the length of the occluder was 5 or 10 deg in different trials). A white vertical bar (height = full height of the screen, width = 0.36 deg) marked the end of the occluder. It corresponded to the target position for the estimation of the TTC (see the Procedure). A schematic representation of the stimuli is provided in Fig. 1.

As for the car object, the speed varied from trial to trial in two levels: 4.31 and 8.62 deg/s. The speed of the PLW also varied from trial to trial in two levels: 7.33 and 10.8 deg/s. The first two rows in Table 1 display the total motion durations of stimuli for each combination of object type, speed, occluder length, and congruency level (i.e., congruent or incongruent motion). Total motion duration encompasses the visible portion of motion (prior to the object disappearing behind the occluder) and the invisible portion (from the moment the object vanishes behind the occluder to when it reaches the white vertical bar with any of its parts). This duration corresponds to the physically correct TTC. Notably, under fixed horizontal speed and occluder length, the PLW and the car exhibited different total motion durations, with the former being longer despite their shared starting position on the screen. Consequently, matching speed levels for both object types would have led to a substantial disparity in total motion durations. Conversely, matching motion durations would have created a significant speed level mismatch. Therefore, we opted to minimize discrepancies in both dimensions simultaneously, even though neither dimension was perfectly matched. We will revisit the issue of differing speeds and motion durations for the two object types in the results section.

**Table 1:** Total, visible, and invisible motion durations (in seconds) categorized by object type (car or PLW), speed (low or high), and occluder length (short or long). It is noteworthy that, for the PLW, all the motion durations differ slightly between congruent and incongruent motions. Notably, motion duration remained independent of motion direction in all instances (i.e., rightwards or leftwards)

		Car				PLW			
		Low speed (4.31 deg/s)	Low speed (4.31 deg/s)	High speed (8.62 deg/s)	High speed (8.62 deg/s)	Low speed (7.33 deg/s)	Low speed (7.33 deg/s)	High speed (10.8 deg/s)	High speed (10.8 deg/s)
		Short occl.	Long occl.	Short occl.	Long occl.	Short occl.	Long occl.	Short occl.	Long occl.
Congruent	Total motion duration	2.297	3.457	1.148	1.728	3.288	4.451	2.328	3.127
Incongruent	Total motion duration	2.297	3.457	1.148	1.728	3.184	4.370	2.293	3.070
Congruent	Visible motion duration	1.137	1.137	0.568	0.568	2.283	2.283	1.612	1.612
Incongruent	Visible motion duration	1.137	1.137	0.568	0.568	2.306	2.306	1.615	1.615
Congruent	Invisible motion duration	1.160	2.320	0.580	1.160	1.005	2.168	0.716	1.515
Incongruent	Invisible motion duration	1.160	2.320	0.580	1.160	0.878	2.064	0.678	1.455

It is important to underline that the length of the occluder corresponded to the distance between its initial position (fixed) and the vertical bar denoting the target location (manipulated). Meanwhile, the distance between the object’s starting point (its center) and the initial position of the occluder remained constant at 7.15 degrees throughout the experiment. The duration of the visible part of the object’s motion (Table 1, third and fourth rows) is computed from the initiation of its movement to the moment the object made contact with the initial position occluder with any of its parts. The interval starting from the latter moment and ending when the object touched the vertical bar (with any of its parts) is defined as the invisible motion duration, or TTC at occlusion (Table 1, fifth and sixth row).

*Procedure*. Before starting the experiment, participants were informed that they would be presented with objects representing a car or a walking person which, at some point of its trajectory, disappeared behind an invisible occluder. They were instructed to press the spacebar at the exact time of contact between the object and the white vertical bar. They were also told to assume that, behind the occluder, the object continued to move as it did before disappearing. They were further instructed that they had to press the spacebar when the target stimulus touched the vertical bar with any of its parts. The object started moving immediately after the start of a trial. After the response, a blank screen appeared 1000 ms, and then a new trial started. No feedback was provided and there was no fixation spot.

We used the psychophysical method of constant stimuli. Trials showing the car and the PLW were presented in separate blocks, administered to the participants in counterbalanced order. Each block consisted of 80 randomly presented trials: 2 object speed × 2 occluder length × 2 motion congruency × 10 repetitions. The motion direction of the object (rightwards or leftwards) was randomized on each trial. Each experimental block was preceded by eight practice trials with no feedback.

## Results

Data can be found on OSF (https://osf.io/tnz7p/?view_only=9b0b49cfec1848eea4082f6c0b4fd1f0). We first identified possible outliers, defined as the participants with an overall mean TE (averaged across all experimental factors) below or above three standard deviations from the mean TE of the respective group (TD or ASD). There was one male outlier in the ASD group (i.e., participant 103 in the original dataset, mean individual TE = 2.572 s, group mean = 0.574 s, *SD* = 0.665 s). The data of this participant were removed and not analyzed further.

We also implemented a data cleaning procedure, wherein trials with a TE deviating more than 2.5 standard deviations from the participant-specific mean TE were excluded from further analysis. These means and standard deviations were calculated individually for each participant, motion congruency condition, object type (car or PLW), object speed, and occluder length. For TD participants, 0.7% of trials were excluded, while for ASD participants, 0.83% were excluded.

The dependent variable was the timing error (TE), defined as the difference between the estimated and the physically correct TTC. The TEs were analyzed using R, version 4.0.4 (R Core Team, 2021). Linear mixed-effects models were employed, utilizing restricted maximum-likelihood (REML) estimation (*lme4* package; Bates et al., 2015). Model comparison was executed through log-likelihood ratio tests using the *anova()* function in the *lmerTest* package (Kuznetsova et al., 2017). The optimal model underwent Type III ANOVA through the *anova()* function in the *lmerTest* package. The Kenward-Roger method to estimate degrees of freedom was used, known for controlling Type I error rates in small samples (McNeish, 2017). Interactions were explored using post-hoc comparisons for linear mixed-effects models (*lsmeans* package, Lenth, 2016) with Kenward-Roger method for the estimation of the degrees of freedom. Tukey’s HSD correction was applied for between-groups comparisons, whereas Bonferroni correction was applied for within-groups comparisons.

### Effects of Motion Congruency, Object Type, and Group

Initial analysis focused on main and interaction effects of motion congruency, object type, and group. Each model featured motion congruency, object type, group, and all the interactions as fixed effects. Differences among models were confined to the random component. Results from models comparisons showed that the optimal model was the one that included random by-subject intercepts and random by-subject slopes for motion congruency and object type. This model significantly outperformed a model that included random intercept and random slope for motion congruency [χ^2^(3) = 744.64, *p* < .001] and a model that included random intercept and random slope for object type [χ^2^(3) = 11.23, *p* = .001]. In turn, the latter two models outperformed a simple model with random by-subject slope, [χ^2^(2) = 7.53, *p* = .023] and [χ^2^(2) = 740.93, *p* < .001] respectively.

ANOVA results showed that the main effects of motion congruency were not statistically significant [*F*(1,29) = 0.34, *p* = .563] due to similar mean TE for congruent motion (*M* = 0.385 s, *SE* = 0.012 s) and incongruent motion (*M* = 0.393 s, *SE* = 0.011 s). No statistically significant interaction effects were observed between motion congruency and group [*F*(1,29) = 0.37, *p* = .551], motion congruency and object type [*F*(1,4828.2) = 2.12, *p* = .146], or motion congruency, object type, and group [*F*(1,4828.2) = 0.17, *p* = .680]. These results indicate that motion congruency had a negligible impact on TE and will not be further considered in subsequent analyses.

Regarding the results concerning object type and group, the main effects of object type were statistically significant [*F*(1,29) = 19.66, *p* < .001], due to larger TE for the car object (*M* = 0.529 s, *SE* = 0.010 s) than for the PLW (*M* = 0.249 s, *SE* = 0.013 s). The main effects of group were not statistically significant [*F*(1,29) = 0.74, *p* = .397], although the mean TE for the ASD group (*M* = 0.449 s, *SE* = 0.013 s) was slightly larger than that for the TD group (*M* = 0.334 s, *SE* = 0.010 s). Importantly, the object type × group interaction was statistically significant [*F*(1,29) = 7.11, *p* = .012]. Post-hoc comparisons showed that, in the case of the car object, the TE was significantly larger for the ASD group (*M* = 0.677 s, *SE* = 0.079) compared to the TD group (*M* = 0.392 s, *SE* = 0.077 s; *p* = .01). In the case of the PLW, no statistically significant differences emerged between the ASD group (*M* = 0.218 s, *SE* = 0.13 s) and the TD group (*M* = 0.278 s, *SE* = 0.12 s; *p* = .732). Further post-hoc comparisons showed that, for TD participants, there was no statistically significant difference between the mean TEs for the car and the PLW (*p* = .204). The latter result is partially inconsistent with the results reported by Mouta et al. (2012) who found less accurate TTC estimations in TD individuals for biological motion than for rigid inanimate motion. Interestingly, for the ASD participants, the TE was significantly larger in the case of the car than in the case of the PLW (*p* < .001).

These results suggest no apparent deficit in TTC estimation for biological motion in ASD participants, which contrasts with previous studies indicating a general deficit in biological motion processing for individuals with ASD. In contrast, ASD participants showed a larger bias than TD participants in the TTC estimation of the car, a scenario involving rigid mechanical motion.

### Analysis of the Possible Confound of Motion Duration

**Fig. 2:**
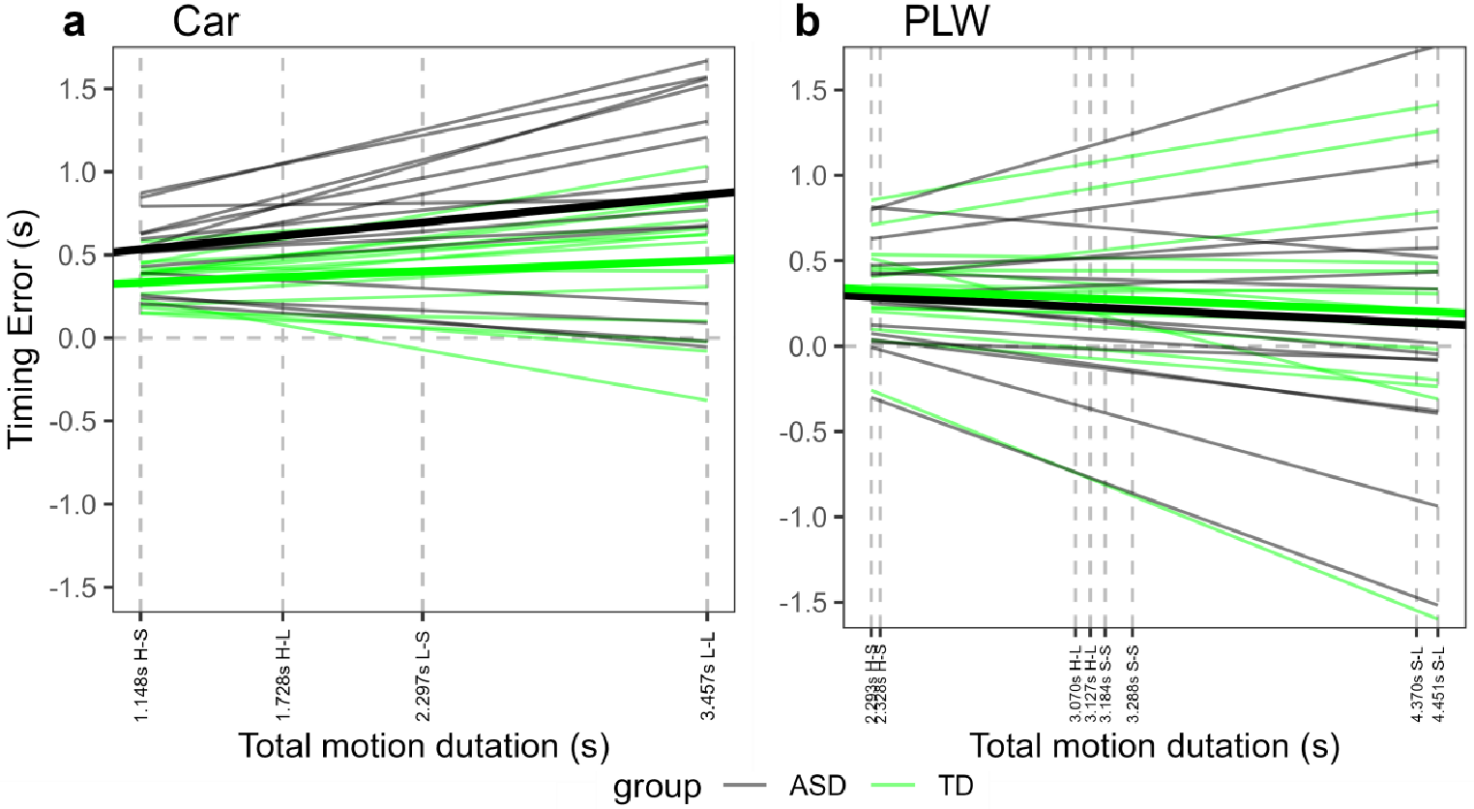
Representation of models predictions for the TE of (a) the car object and (b) the PLW. In both panels, thick lines represent estimated fixed effects, while thin lines represent estimated random effects. Dashed vertical lines correspond to the motion duration for each combination of occluder length and speed (refer to Table 1 for details). On the horizontal axis, each motion duration is presented alongside two letters. The first letter denotes the object’s speed (H = high, L = low), and the second letter denotes the length of the occluder (L = long, S = short). In the case of the PLW (panel b), adjacent vertical lines pertain to incongruent motion (left) and congruent motion (right)

It is worth underlining that the car object exhibited shorter total motion durations compared to the PLW (see Table 1). Consequently, it is arguable that the performance difference between the ASD and TD groups across both object types may primarily relate to motion durations rather than the object type itself. Specifically, the ASD group’s challenge in estimating the TTC of the car object could be attributed to a specific difficulty in TTC estimation for brief motion durations compared to TD participants, with no comparable difficulty for long motion durations. To further illuminate this matter, we analyzed TE variations by group and total motion duration separately for the car and the PLW. The best fitting linear mixed-effects models are represented in Fig. 2. A representation of raw individual means can be found on OSF (https://osf.io/tnz7p/?view_only=9b0b49cfec1848eea4082f6c0b4fd1f0). Specifically, Supplementary Figs. 1 and 2 on OSF represent the mean individual TEs as a function of motion duration and group, for the car object and the PLW, respectively. Furthermore, Table 2 represents the mean TE for the two groups, for each combination of object type, occluder length, object speed, and motion congruency. These were the variables that determined the total motion duration. A graphical representation of individual mean TE as a function of group, occluder length, object speed, and motion congruency is available in Supplementary Fig. 3 (car object) and 4 (PLW) on OSF. Furthermore, a graphical representation of mean individual estimated TTC (rather than TE) as a function of group, occluder length, object speed, and motion congruency is available in Supplementary Fig. 5 (car object) and 6 (PLW) on OSF.

**Table 2:** Mean TE with standard error (in seconds) for each combination of group, object type, speed, occluder length and motion congruency

			TD		ASD	
			Car	PLW	Car	PLW
Low speed	Short occluder	Congruent	0.627 (0.052)	0.332 (0.101)	0.944 (0.097)	0.265 (0.106)
		Incongruent	0.604 (0.047)	0.439 (0.093)	0.918 (0.097)	0.328 (0.098)
	Long occluder	Congruent	0.403 (0.110)	0.133 (0.190)	0.747 (0.167)	0.085 (0.231)
		Incongruent	0.384 (0.105)	0.176 (0.173)	0.785 (0.162)	0.139 (0.194)
High speed	Short occluder	Congruent	0.321 (0.030)	0.276 (0.072)	0.472 (0.060)	0.267 (0.090)
		Incongruent	0.325 (0.034)	0.209 (0.066)	0.473 (0.052)	0.234 (0.081)
	Long occluder	Congruent	0.242 (0.060)	0.360 (0.131)	0.545 (0.100)	0.211 (0.173)
		Incongruent	0.232 (0.067)	0.330 (0.129)	0.529 (0.091)	0.216 (0.168)

For the car object analysis, we first compared two linear mixed-effects models. Both models included total motion duration, group, and their interaction as fixed effects. The first model featured a random by-subject intercept, while the second included a random by-subject intercept and a random by-subject slope for total motion duration. The log-likelihood ratio test indicated that the latter model significantly outperformed the former [χ^2^(2) = 411.65, *p* < .001]. The model predictions are represented in Fig. 2a. ANOVA results revealed that the main effect of total motion duration was statistically significant [*F*(1,29) = 10.75, *p* = .002]. This main effect quantifies the impact of total motion duration on TE for the ASD group. Specifically, the TE tended to increase with motion duration for the ASD group (*b* = 0.143, *SE* = 0.044). The total motion duration × group interaction was not statistically significant [*F*(1,29) = 1.91, *p* = .178], indeed the TE tended to increase with motion duration for TD participants as well (*b* = 0.058, *SE* = 0.062). Post-hoc comparisons demonstrated that TE for the ASD group was significantly greater than TE for the TD group at the first three levels of motion durations characterizing the car object (*p*s < .025), whereas the difference did not reach a statistical significant level for the longest motion duration (*p* = .064).

Regarding the PLW analysis, we followed the same procedures as for the car object. A log-likelihood ratio test indicated that a model with total motion duration, group, and their interaction as fixed effects, along with by-subject intercept and by-subject slope for total motion duration as random effects, provided a significantly better fit than an equivalent model without a by-subject random slope [χ^2^(2) = 355.4, *p* < .001]. ANOVA results revealed that the main effect of total motion duration, reflecting its effects on TE for the ASD group, was not statistically significant [*F*(1,29) = 2.32, *p* = .138]. The total motion duration × group interaction was also not statistically significant [*F*(1,29) = 0.02, *p* = .896], indicating similar estimated fixed linear regression coefficients for the ASD group (*b* = -0.073, *SE* = 0.117) and the TD group (*b* = -0.061, *SE* = 0.088). Post-hoc comparisons demonstrated that TEs for the ASD and TD groups were not significantly different at each of the eight total motion durations characterizing the PLW, represented by vertical lines in Fig. 2b, *p*s > .44.

As anticipated, one potential explanation for the observed difference between ASD and TD participants in the car object analysis (but not in the PLW analysis) was the possibility of a specific deficit in TTC estimation for short motion durations among ASD participants. However, this hypothesis was not supported by the data. For the ASD group, the TE increased with motion duration for the car object, whereas the TE did not vary in a statistically significant manner for the PLW. Furthermore, if the hypothesis were valid, we would expect the TE difference between the two groups to diminish as motion duration increased, which contradicted the observed results. It is also worth noting that the longest motion duration for the car object exceeded the shortest motion duration for the PLW. If the disparity in TE between the ASD and TD groups were solely influenced by motion duration, one might anticipate a more substantial between-group difference for the shortest PLW duration compared to the longest car object duration. However, a visual examination of Fig. 2a and b clearly demonstrates that this was not the scenario. The same logic applies to stimulus speed. Given that the maximum speed for the car object exceeded the minimum speed for the PLW (Table 1), a specific deficit in ASD participants with low speeds should result in a smaller between-group difference for the car at high speed compared to the PLW at low speed. However this was not the case.

In summary, the results do not substantiate the hypothesis that motion duration played a pivotal role in mediating the difference between ASD and TD participants across the two object types. Instead, it appears that inherent characteristics of the two object types can account for this distinction.

Motion duration fails to account for between-group differences in relation to object type. Despite this, exploring how differences in motion duration, manipulated through object speed and occluder length, influenced the TE is of interest. Previous studies suggest that an overestimation of TTC (i.e. positive TE) is likely for short occlusion durations, while underestimation (i.e. negative TE) is probable for long occlusion durations (Battaglini & Mioni, 2019; Bennett et al., 2010; Makin, 2018; Tresilian, 1995). However, our findings deviate from this, as no evidence of TTC underestimation was observed, even for the shortest motion/occlusion duration involving the high-speed car object with a short occluder (Table 2 and Supplementary Figs. 3 to 6). Participants consistently overestimated TTC in all experimental conditions. Notably, a consistent overestimation tendency emerged in previous studies involving TD participants estimating the TTC of a vertically falling object (Vicovaro et al., 2019, 2021).

Regardless of TE sign, assessing whether TE decreased with motion/occlusion duration, as reported in previous TTC studies, remains inconclusive. There was a general trend for TE to be smaller with the long occluder compared to the short occluder (Table 2), aligning with the hypothesis of a negative relationship between occlusion duration and TE due to longer motion/occlusion duration with the long occluder. However, this relationship reversed for the PLW moving at high speed for TD participants and for the car object moving at high speed for ASD participants. Additionally, contrary to the anticipated direction, TE tended to be larger for low speed compared to high speed, except for the PLW with the long occluder. These results underscore the notion that TE likely depends on various situational factors, preventing general predictions. The unique results may be attributed to the specific nature of the objects involved—PLW and a car representation—distinct from the simpler geometrical shapes typically used in TTC studies.

## Discussion

The ability to predict the trajectory of a moving target is crucial in our daily life activities, for instance, it allows us to catch moving objects or drive safely. In our study, participants performed a TTC estimation task, where a moving object passed behind an occlusion, and the participants had to press a button (spacebar) when they believed that the occluded object had reached the target position. Within the paradigm, we considered two types of moving stimuli, a car and a PLW, along with factors that could potentially affect the individual’s performance (i.e. total motion duration, object speed, length of the occluder, and movement congruency). The focus of our research was to compare the performance of young adults with ASD and TD, since the literature reports a wide range of atypical perceptions within the ASD population, but, to our knowledge, TTC estimation has not been investigated to date.

Contrary to our expectations, we did not find an overall larger TE for ASDs compared to TDs. Specifically, our results indicated a significantly lower performance when TTC estimation involved a car, but comparable performances between groups when the task involved a PLW. Some studies suggest that ASD individuals, even in the presence of adequate intellectual abilities, are characterized by greater slowness in performing some cognitive tasks, especially those requiring cognitive flexibility, planning, sustained attention, and processing speed (Fried et al., 2016; Haigh et al., 2018). However, it is unlikely that our result may reflect a general deficit in attentional resources, processing speed or temporal synchrony in ASDs, otherwise we would have observed generally larger TEs for ASDs regardless of stimulus type.

The difficulties in estimating the TTC for the car condition in our ASD sample are consistent with the literature, as ASDs tends to exhibit an atypical perception of moving stimuli (Dakin & Frith, 2005) and in global motion (Vandenbroucke et al., 2008). We can hypothesize that the tendency of ASD participants to systematically overestimate the TTC of the car object with respect to the TD participants is related to the fact that the car condition in our task involved rigid motion that requires global rather than local processing. Several studies have shown that ASD individuals are more accurate and faster at processing local elements of stimuli rather than global structure (Bertone et al., 2003; Caron et al., 2006; Spencer et al., 2000). Furthermore, according to the weak central coherence (WCC) theory (Frith, 1989; Frith & Happé, 1994), ASD individuals have a tendency to pay more attention to local details, rather than to global form and meaning (Baron-Cohen et al., 2001). WCC theory could account for superior performance in tasks for which locally oriented processing is advantageous, and it can also account for lower performance in tasks in which a holistic or global integration of visual features is required (Bertone et al., 2003). As estimating the TTC of the car object involved global motion processing, the WCC theory can account for ASDs’ poorer performance with respect to TDs. This aspect could have important implications and needs further consideration as a future research. Since TTC estimation is engaged in a wide range of daily activities, it would be interesting to understand the ecological impact of the atypical performance of ASDs. For instance, it would be interesting to understand if this outcome is associated with driving skills, which is a daily situation where TTC estimation is embedded in decision-making and executive processes.

Results regarding the PLW condition are interesting, as we did not find differences in TEs between the ASD and the TD group. We should also note that, even if not at a statistically significant level, ASD performance was on average better than TD. Moreover, while no statistically significant difference emerged between the TEs for the car and the PLW in the case of the TD group, ASDs exhibited significantly smaller TE for the PLW compared to the car. We could suggest some hypotheses which could explain these results. First, we do not exclude that the tendency to focus on the local at the expense of the global may have been an optimal strategy for ASDs in the case of the TTC estimation for the PLW. It has already been reported that adults with ASD could achieve comparable outcomes to a control group in tasks related to biological motion, which has been considered due to different underlying neural processes (Freitag et al., 2008; McKay et al., 2012). Moreover, differences between ASDs and TDs in interpreting biological motion seem to decrease with the increase of participants’ age (Federici et al., 2020; Todorova et al., 2019). Since our ASD sample was composed of young adults our results could further support this evidence. Recent studies show that differences between TD and ASD in biological motion perception are evident when the task requires perceiving emotions but not for motion perception per se (Foglia et al., 2022; Todorova et al., 2019). This would suggest that biological motion perception in TD individuals relies on specialized mechanisms for social perception, whereas ASD individuals may employ alternative strategies (Foglia et al., 2022). In this regard, it could be hypothesized that ASD individuals might employ alternative cognitive and perceptual strategies to process biological stimuli compared to TD individuals. For example, studies based on connectivity analysis have shown that TD individuals use brain regions consistent with form and movement integration patterns. In contrast, ASDs show distinct networks for form and movement, suggesting independent processing (McKay et al., 2012).

### Limitations and Conclusions

Despite the interesting findings, our study presents some limitations. Due to the limited availability of ASD participants, the sample size was relatively small, thus future studies should try to replicate our findings considering larger groups. TD participants were recruited from a local University, thus this sampling strategy could limit the generalizability of our findings. Our ASD sample was composed of a higher prevalence of males, however, this issue is frequently faced in ASDs recruitment as the disorder affects about four males for every one female (Valenti et al., 2019). In a future study, it would be interesting to compare the performance between males and females from an ASD sample. We did not focus on other variables which could potentially be associated with the performance, for instance it would be interesting to consider attention levels and visual acuity. In a future study, it would be useful to incorporate an initial control condition in order to obtain baseline reaction times. Moreover, all of our ASD participants were level-1 without ID, we choose this sample to avoid possible effects due to difficulties in understanding the task, however, most of the ASD population presents cognitive impairments or other concomitant disorders, thus it would be interesting to explore TTC estimation skills in more common ASD profiles. On the other hand, this choice was also made as it allowed the detection of differences due to the ASD condition per se, controlling for possible confounding variables.

In conclusion, ASDs showed an atypical performance in TTC estimation. Because this skill is involved in a wide range of daily contexts, it is important to further explore the impact that it could represent for this population, maybe in a more natural context. Indeed, the outcomes of our study necessitate cautious interpretation regarding their applicability to real-world situations, given the unique characteristics of the experimental conditions presented to the participants, such as reduced speed and limited spatial shifts in the stimuli compared to real-life scenarios. Biological motion represents an important feature that could affect the TTC estimation processes. The nature of this effect still needs to be uncovered, specifically, we suggest that future studies should consider possible advantages and disadvantages that this type of stimulus could represent during other motion extrapolation processes.
